# Supplementary material for: Cumulative Weighing of Time in Intertemporal Tradeoffs
Source: J Exp Psychol Gen. 2016 Sep;145(9):1177–205. doi: 10.1037/xge0000198 (PMC4998108; doi:10.1037/xge0000198)
Supplement: Supplementary file 1 [file Scholten_Individuals.zip › plots/e29_p462_eg2_priors_and_posteriors.pdf]

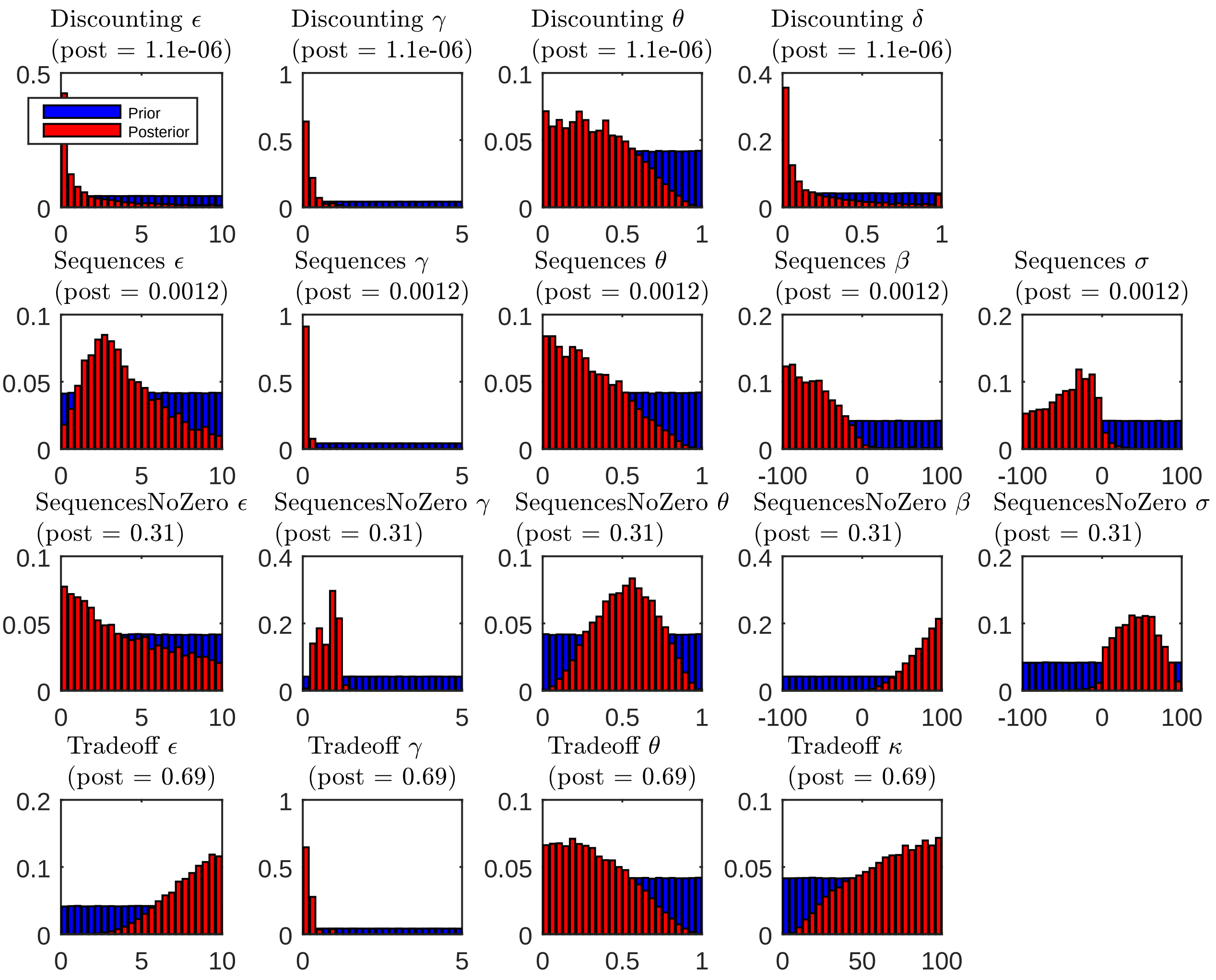
Discounting ϵ
(post = 1.1e-06)
Prior
Posterior
Discounting γ
(post = 1.1e-06)
Discounting θ
(post = 1.1e-06)
Discounting δ
(post = 1.1e-06)
Sequences ϵ
(post = 0.0012)
Sequences γ
(post = 0.0012)
Sequences θ
(post = 0.0012)
Sequences β
(post = 0.0012)
Sequences σ
(post = 0.0012)
SequencesNoZero ϵ
(post = 0.31)
SequencesNoZero γ
(post = 0.31)
SequencesNoZero θ
(post = 0.31)
SequencesNoZero β
(post = 0.31)
SequencesNoZero σ
(post = 0.31)
Tradeoff ϵ
(post = 0.69)
Tradeoff γ
(post = 0.69)
Tradeoff θ
(post = 0.69)
Tradeoff κ
(post = 0.69)
